# Surface-Tension-Confined Channel with Biomimetic Microstructures for Unidirectional Liquid Spreading

**DOI:** 10.3390/mi11110978

**Published:** 2020-10-30

**Authors:** Yi Zhang, Yang Gan, Liwen Zhang, Deyuan Zhang, Huawei Chen

**Affiliations:** 1 School of Mechanical Engineering and Automation, Beihang University, Beijing 100191, China; zhangyi92@buaa.edu.cn (Y.Z.); ganyang2018@buaa.edu.cn (Y.G.); lwzhang@buaa.edu.cn (L.Z.); zhangdy@buaa.edu.cn (D.Z.); 2 Beijing Advanced Innovation Center for Biomedical Engineering, Beihang University, Beijing 100191, China

**Keywords:** unidirectional liquid spreading, liquid manipulation, biomimetic microstructures, surface-tension-confined channel

## Abstract

Unidirectional liquid spreading without energy input is of significant interest for the broad applications in diverse fields such as water harvesting, drop transfer, oil–water separation and microfluidic devices. However, the controllability of liquid motion and the simplification of manufacturing process remain challenges. Inspired by the peristome of *Nepenthes alata*, a surface-tension-confined (STC) channel with biomimetic microcavities was fabricated facilely through UV exposure photolithography and partial plasma treatment. Perfect asymmetric liquid spreading was achieved by combination of microcavities and hydrophobic boundary, and the stability of pinning effect was demonstrated. The influences of structural features of microcavities on both liquid spreading and liquid pinning were investigated and the underlying mechanism was revealed. We also demonstrated the spontaneous unidirectional transport of liquid in 3D space and on tilting slope. In addition, through changing pits arrangement and wettability pattern, complex liquid motion paths and microreactors were realized. This work will open a new way for liquid manipulation and lab-on-chip applications.

## 1. Introduction

Unidirectional liquid spreading has attracted worldwide attention due to its extensive application prospect in diverse fields, such as water harvesting [1,2,3], self-lubrication [4], liquid separation [5], and microfluidic operation [6,7,8]. However, the existing liquid manipulation methods induced by external driving fields are gradually unable to meet the requirements of green, efficient and portable microfluidic devices [9,10,11]. In past years, different strategies have been proposed to break the equilibrium state of droplet on surfaces and achieve self-driven asymmetric spreading. Through chemical modulation of surface wettability, liquid can move to a preferential direction due to imbalanced surface tension forces [8,12,13,14]. Moreover, liquid flow control can be realized by constructing structurally modified surfaces with deflected nano-pillars [15], triangular posts [16] and fin-like structures [17]. Despite extensive progress, the limited transport distance and relatively simple transport route are far from what would be demanded for practical applications.

**Figure 1:**
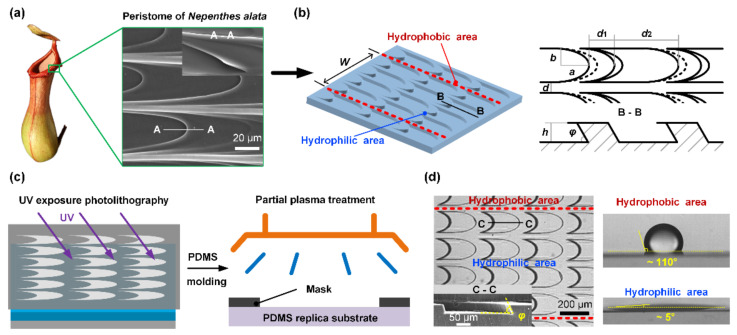
(**a**) Multilevel structures of the natural peristome surface of *Nepenthes alata*, involving hierarchical microgrooves and duck-billed microcavities. (**b**) Design of the surface-tension-confined (STC) unidirectional liquid spreading channel and biomimetic microcavities. (**c**) Schematic of the channel fabrication process. (**d**) Characterization of the STC unidirectional liquid spreading channel.

Inspirations from nature offer a biomimetic way to develop functional materials. In order to adapt to the complex living environment, many plants and animals have evolved intriguing organs composed of hierarchical micro-nano structures, which process unique wetting properties. For example, Small drops in mist can also be captured by the periodic spindle-knot structures of spider silk, then move directionally from joints to knots as water condenses [18]; cactus spines are capable of collecting water from air and transporting water directionally by means of the conical trichomes [1]; capillary channels network between the overlapped scales of Texas horned lizard enables water to transport towards its snout preferentially [19]. Recently, a new mechanism of autonomous unidirectional water transport discovered on the peristome surface of *Nepenthes alata* provides more options for the design of anisotropic wetting surface [20]. The hierarchical microgrooves, unique duck-billed contour, and wedge corner of microcavities have been proven to be key factors in the unidirectional transport of liquid with a fast speed (Figure 1a). Through mimicking its multilevel structures, several artificial peristome-mimetic surfaces have been successfully fabricated via two-step inclined photolithography [21], stereo-lithography [22], and ferrofluid assembly [23]. However, how to improve the stability of pinning effect, and, especially, simplify fabrication process are still daunting challenges.

Herein, inspired by the peristome surface of *Nepenthes alata*, an easy-fabricated surface-tension-confined (STC) channel with biomimetic microstructures is proposed to realize unidirectional spreading. The structured substrate with arc-shaped pits array are fabricated via one-step photolithography and polydimethylsiloxane (PDMS) replica molding. Through partial plasma treatment, hydrophilic–hydrophobic pattern is constructed on the substrate to form a STC channel. When liquid transports on the channel, the lateral flow of liquid is confined by the hydrophobic boundary and the reverse flow is prohibited by the arc-shaped microcavities, thus ensuring that the liquid is transmitted only in the desired direction. Through comparing the liquid transport behavior on STC unidirectional liquid spreading channel, the effect of channel parameter and microcavities structural features on anisotropic liquid spreading is explored systematically, and the stability of pinning ability is demonstrated. The microscopic propagation process of liquid advancing edge is observed and the underlying mechanism of unidirectional liquid spreading is further validated. Finally, spatial and antigravity unidirectional transport, complex liquid manipulation, and multiphase flow reactions are realized easily on the designed channels.

## 2. Materials and Methods

### 2.1. Materials

Permanent epoxy negative photoresist SU-8 2015 and developer were purchased from MircoChem, Newton, MA, USA. Polydimethylsiloxane (PDMS, sylgard 184) was purchased from Dow Corning, Midland, MI, USA and was used as-received. Hollowed-out 304L stainless mask was fabricated by Precise Corporation (Shenzhen, China). Isopropyl alcohol was purchased from Shanghai Macklin Biochemical Co, Ltd. Aniline blue, ammonium thiocyanate (NH_4_SCN, reagent grade 97.5%) and ferric chloride (FeCl_3_, reagent grade 97%) were obtained from Sigma Aldrich. De-ionized water was obtained from an Ultrapure Water Polishing System (CANSHI, Ningbo, China).

### 2.2. Design and Fabrication of STC Channel with Biomimetic Microstructures

In order to reduce the complexity in manufacturing the multilevel structures of artificial peristome surfaces, we proposed a simple method to construct the unidirectional liquid spreading channel that combines the striped wettability pattern and microstructures as shown in Figure 1b, where *W* is the width of hydrophilic stripe. The designed biomimetic microcavities contain two main typical structural features, including the elliptic edge and the wedge angle. Based on the line-width precision of the photolithography film mask, the semi-major axis length *a* and semi-minor axis length *b* of ellipse arc are 160 μm and 80 μm, respectively. The length of stage *d*_1_ and pit *d_2_* are 120 μm and 200 μm, respectively. The distance *d* between two pits in width direction is 40 μm. The wedge angle *φ* depends on the tilt exposure angle.

The negative template was fabricated by UV exposure photolithography (Figure 1c). SU-8 2015 photoresist was firstly coated on the cleaned glass substrates by a spin coater (KW-4T, IMECAS, Beijing, China) with a rotational speed of 1500 rpm for 30 s, the thickness of the coating is about 30 μm. Then the soft bake of the substrate was conducted on a hot plate (KWH-600, CHEMAT, Los Angeles, CA, USA) in two steps: the substrate was baked at 65 °C for 3 min and subsequently at 95 °C for 5 min. UV exposure was performed after the soft bake under a UV source (wavelength is 365 nm and irradiation intensity is 16 mW/cm^2^) with a certain angle for 20 s as shown in Figure 1c. The post exposure bake process was executed with the same steps as the soft bake process. After developing in SU-8 developer for 5 min and rinsing with deionized water and isopropyl alcohol for 10 s successively, the negative template with inclined micro-pillars was prepared. The relative humidity in the experiments was kept constant at ~40%, and the room temperature was ~20 °C.

Replica molding provides an efficient and low-cost method for fabricating microstructures, and different polymers such as polydimethylsiloxane (PDMS) [24], styrenic block copolymers (SBS) [2], cyclic olefin copolymer (COC) [25], and poly(vinyl alcohol) (PVA) are widely used [22]. Here, we used PDMS as the replication material to obtain textured surfaces, due to its biocompatibility, optical transparency and ease of molding [26,27,28]. PDMS and curing agent were mixed at a mass ratio of 10:1, well stirred for 10 min and degassed in a vacuum oven for 20 min. Subsequently, the prepolymer was casted onto the negative template and degassed again. After curing the sample at 75 °C for 40 min and demolding, the PDMS replica substrate with inclined microcavities was obtained. The SEM image in Figure 1d shows the cross-section of the microstructures. In order to fabricate the STC channel, partial air plasma treatment (YZD08-2C, SAOT (Beijing) Tech Co, Ltd., Beijing, China) was performed on the PDMS replica covered with a hollowed-out stainless mask, the treatment process was conducted at an RF power of 160 W and RF frequency of 40 KHz for 20 s, a system pressure of 100 μbar, and a flow rate of 0.3 L/min. Wettability was measured by depositing 4 μL water on the surface by an optical angle measuring system (SL200B, Solon, Zhengzhou, China). The exposed part becomes hydrophilic and the contact angle is about 5°, whereas the unexposed part remains hydrophobic and the contact angle is about 110°. The relative humidity in the experiments was kept constant at ~40%, and the room temperature was ~20 °C.

### 2.3. Observation of Liquid Transport Behavior

The channel surface was placed on a LED backlight (HF-FX100, Lemons, Dongguan, China). Liquid (aqueous solutions of Aniline blue) was dripped on the hydrophilic area of channel through a capillary tube with the aid of a microliter syringe (100 μL, GAOGE) and a micro injector (RSP01-BG, RISTRON, Jiaxing, China). The liquid transport behavior was recorded by a high-speed camera (I-speed LT, Olympus, Tokyo, Japan) at 1000 fps and a digital camera (EOS 800D, Canon, Tokyo, Japan) at 50 fps. The microscopic liquid transport process was recorded under a microscope (BX51, Olympus, Tokyo, Japan).

## 3. Results and Discussion

### 3.1. Liquid Transport Behavior on STC Unidirectional Liquid Spreading Channel

**Figure 2:**
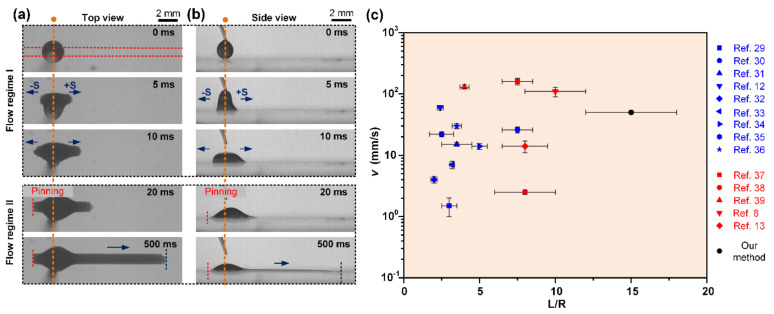
In situ observation of liquid transport behavior on STC channel from (**a**) the top view and (**b**) the side view. The liquid transport can be identified as two flow regimes: Flow regime I: bidirectional spreading; Flow regime II: unidirectional spreading. The red and orange dashed lines denote the hydrophobic boundary and the position of dripping liquid, respectively. (**c**) Comparison of spreading velocity and normalized distance L/R (L is the transport distance of a droplet in the forward direction and R is the droplet radius) among different surfaces with gradient (blue symbols) or patterned (red symbols) wettability.

As shown in Figure 2 and Supplementary Materials Video S1, the time-lapse liquid transport process on the STC channel is captured from the top view and side view, where the liquid transport can be clearly identified as two flow regimes: bidirectional spreading and unidirectional spreading. Here, the arc edge convex side is defined as the positive direction (+S) and its opposite side as the negative direction (−S). After dripping liquid on the hydrophilic area of STC channel, the droplet tends to spread bidirectionally under inertia during the initial droplet–substrate contact process (Figure 2a). As time persists, the second regime emerges, liquid is quickly pinned in the -S direction within tens of milliseconds. Then the liquid spreads unidirectionally along the hydrophilic stripe until a slender liquid trip is formed, and the rear side of liquid remains static throughout the second flow regime (Figure 2b). Compared with the reported unidirectional liquid spreading surfaces with gradient ([12,29,30,31,32,33,34,35,36]) or patterned ([8,13,37,38,39]) wettability, long-range and fast liquid self-driven transport was realized on the designed STC channel, which illustrates the advantages of our method (Figure 2c).

### 3.2. Impact of Channel Parameters on Unidirectional Spreading

**Figure 3:**
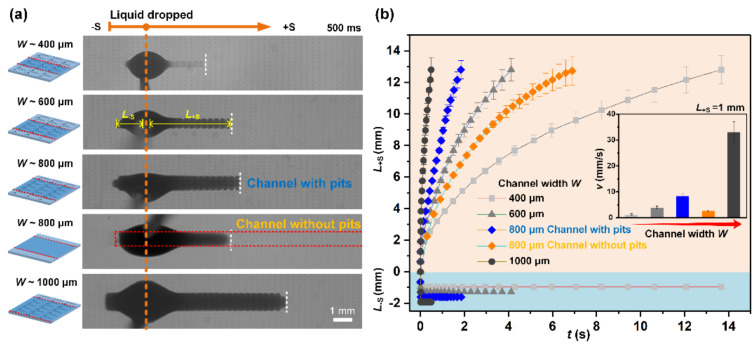
(**a**) In situ observation of liquid transport on STC channels with different hydrophilic stripe widths at a time of 500 ms. (**b**) Transport distance of liquid as function of time. *L*_+S_ and *L*_−S_ are the liquid transport distances in +S and −S direction respectively at same time interval. The inset image shows the average transport velocity of liquid on the channels with different widths.

To explore the impact of channel parameter on liquid spreading behavior, a variety of STC unidirectional liquid spreading channels with different hydrophilic stripe width, as shown in Figure 3a, were fabricated through changing the hollowed-out shape of stainless mask, where the structural features of pits array on the bottom surface remain the same. The volume of dropped liquid Ω is kept proportional to the width of channels, which is approximately 2, 3, 4, and 5 μL for the channels with the width of 400, 600, 800, and 1000 μm, respectively, to maintain the same characteristic geometric length ${\left(\Omega /W\right)}^{0.5}$ of the droplet [40]. Apparent disparities of the liquid transport in the +S direction appeared at 500 ms after dropping the liquid, where the time-dependent transport distance in +S direction (*L*_+S_) and −S direction (*L*_−S_) were plotted in Figure 3b. The liquid transport velocity in the +S direction gradually declines with the increase in *L*_+S_. With channel width increasing from 400 μm to 1000 μm, the average speed increases from ~1.2 mm s^−1^ to ~33.5 mm s^−1^ at a distance of 1 mm (*L*_+S_ = 1 mm). Notably, liquid travels faster in the channel with pits on bottom surface than in a smooth channel of the same width, the average speed is only about 2.6 mm s^−1^ for 800 μm smooth channel, indicating that the pits array can enhance the spreading ability. The rear direction of smooth channel maintains hydrophobic to ensure that liquid is only transported towards the +S direction. In the −S direction, the pinning effect is basically consistent among these channels and reverse flow only happens in the first few milliseconds.

### 3.3. Underlying Mechanisms of Unidirectional Liquid Spreading

#### 3.3.1. Mechanism for Liquid Spreading

**Figure 4:**
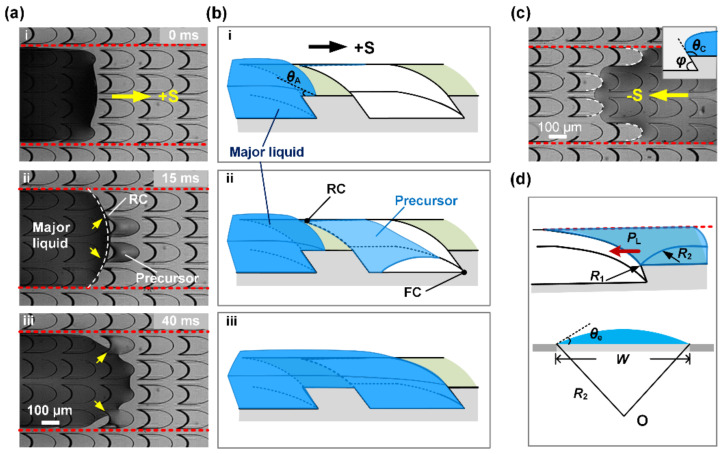
(**a**) Time-dependent microscopic propagation behavior of liquid advancing edge. (**b**) Schematic depiction of the liquid forward flow. $\theta_{A}$ is the advancing angle. rear corner (RC) and front corner (FC) represent rear corner and front corner of microcavity, respectively. (**c**) Morphology of liquid film on the stage in -S direction. $\theta_{C}$ is the critical contact angle at which the liquid diffuses downward along the arc-shaped edge. (**d**) Schematic drawing of the 3D morphology of liquid rear side. $P_{L}$ is the Laplace pressure of liquid surface. $R_{1}$ and $R_{2}$ are the radii of curvature of liquid film in horizontal and vertical section, respectively. $\theta_{e}$ is the intrinsic contact angle.

To reveal the mechanisms of anisotropic liquid transport, the microscopic liquid spreading process was recorded as shown in Figure 4. The shape of the advancing edge of major liquid is convex under the restriction of the hydrophobic boundaries on both sides (Figure 4a, i). Liquid propagation process starts with the contact of advancing edge on the sharp rear corner (RC) of central pits of the channel, the pits are rapidly filled from the rear corner to front corner (FC) by liquid precursor and air is squeezed out (Figure 4a, ii). Then the major liquid quickly spreads forwards and coalesces the precursor. As shown in Figure 4a, iii, the pits near the boundaries are also gradually filled from RC to FC until the advancing edge of the major liquid climbs onto the stage to continue filling next structures in +S direction.

For the liquid forward spreading, quantitative estimate for the significance of gravity and surface tension forces can be scaled by Bond number, $Bo=\rho gR^{2}/\gamma$, where ρ is the density of liquid, g is the gravitational acceleration, *R* is the radius of the droplet and γ is the surface tension. In our experiments, the Bond number ranges between 0.08 and 0.17 for the droplets. Since Bo<1, the gravity effect can be negligible [41].

The flow dynamics of the liquids on the STC unidirectional liquid spreading channel show a dependence on the channel width, which is similar to that on smooth STC channel [42]. An increase in channel width results in a significant increase in speed. The difference in liquid spreading velocity on smooth and structured STC channel can be explained from two aspects. On the one hand, as shown in Figure 4b, i, the advancing angle $\theta_{A}$ of liquid on the stage approximates to zero due to the complete hydrophilic property of the STC channel. Affected by the strong capillary rise in RC, the precursor is first imbibed into microcavities before the movement of the major liquid film, and the forward transport is enhanced obviously compared to liquid wetting on smooth channel owing to the transition of liquid/solid interface to liquid/liquid interface (Figure 4b, ii,iii) [43]. The liquid imbibition and filling process in the microcavity can be simplified to the capillary flow in an open channel with wedge corners, which are relevant to the pit dimensions and the wedge angle of front corner [44,45].

On the other hand, the liquid wetting state on rough solid is Wenzel state, which means the apparent contact angle channel $\theta_{a}$ and intrinsic contact angle $\theta_{e}$ yields to Wenzel’s relation [46]

(1) $$\cos\theta_{a}=r\cos\theta_{e}$$

where *r* is the ratio of actual area to projected area. For hydrophilic rough surface, we have $\theta_{e}<\pi /2$ and r>1, which implies $\theta_{a}>\theta_{e}$. A more wettable surface is formed due to the roughness, leading to a faster liquid transport speed on the structured STC channel.

#### 3.3.2. Mechanism for Liquid Pinning

In the −S direction, reverse flow is prevented by microcavities and the three-phase contact line (TCL) is along the sharp edge (Figure 4c). As the inset image shown in Figure 4c, when liquid is able to spread spontaneously down the edge, the critical contact angle *θ*_c_ is equal to $180{}^{\circ}-\varphi +\theta_{e}$ [47], which is larger than the advancing contact angle $\theta_{A}$. It is noted that a larger wedge angle φ causes a larger critical contact angle in the +S direction, therefore, liquid spreads towards the +S direction preferentially and pins in the −S direction. Previous study indicates that groove structure can enhance transport ability, however, it impedes the unidirectional liquid spreading control due to the strong capillary rise effect in the corner, where the pinning effect is easy to collapse [21,48,49]. Nevertheless, when the physical sidewalls of the groove were replaced by chemical boundary, in which the resistance of the hydrophobic boundary induces a channel width independent force [50], excellent unidirectional liquid spreading performance was achieved and reverse flow only happened in the initial stage under the influence of inertia.

Apart from the critical contact angle, the additional pressure generated by concave liquid surface likewise plays an important role in the pinning effect. According to the Young–Laplace equation,

(2) $$P_{L}=\gamma \left(R_{1}^{-1}+R_{2}^{-1}\right)$$

where *P*_L_ is the Laplace pressure of liquid surface, *R*_1_ and *R*_2_ represent the principal radii of curvature of the liquid surface in horizontal and vertical section, respectively (Figure 4d). In the horizontal section, the morphology of liquid film is ellipse, thus *R*_1_ is calculated as $R_{1}=b^{2}/a$. For the microcavity with a semi-major axis length of 160 μm and a semi-minor axis length of 80 μm, *R*_1_ = 40 μm. In the vertical section, *R*_2_ related to the channel width and intrinsic contact angle is estimated as $R_{2}\sim W/2\sin\theta_{e}$; the value of *R*_2_ ranges between 1 mm and 5 mm in these experiments, which is an order magnitude larger than *R*_1_. Consequently, the Laplace pressure is negative and mainly depends on *R*_1_.

### 3.4. Impact of Microcavity Structural Features

#### 3.4.1. Influence of Microcavity Wedge Angle

**Figure 5:**
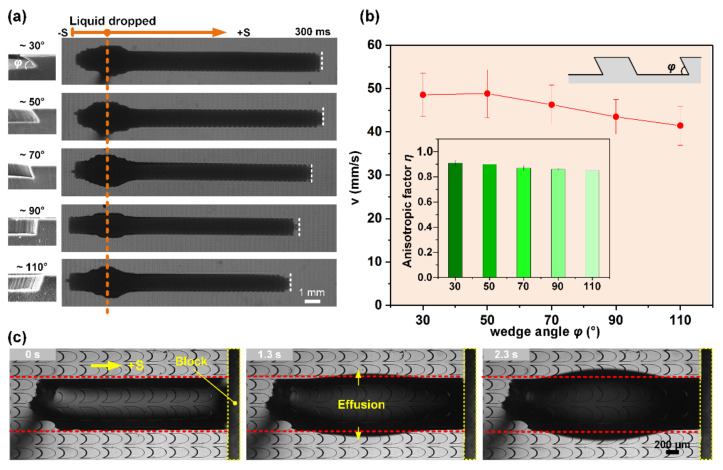
(**a**) In situ observation of STC unidirectional liquid spreading channels with different wedge angle ranging from about 30° to 110° at a time of 300 ms. (**b**) The velocity and anisotropic liquid spreading factor in different structures. (**c**) Time-lapse images of pinning failure process.

For artificial peristome surfaces, the wedge angle is a key factor for impeding liquid back flow, and greatly increases the complexity of fabricating the inclined microcavities [40,48,51]. Here, a strong pinning stability was demonstrated on the designed STC unidirectional liquid spreading channel, which showed a potential in constructing low-cost microfluidic platforms and microreactors. We fabricated a series of channels with different wedge angles that ranges between 30° and 110°, through changing the included angles between the SU-8 substrate and UV source during exposure to explore the effect of wedge angle on the transport of the liquid on STC channels (Figure 5a). The widths of hydrophilic stripes are all about 1.2 mm. The right side of Figure 5a displays the liquid spreading state at 300 ms after equivalent droplets were deposited on the as-fabricated channels. In the +S direction, the pinning ability is basically constant, the spreading distance $L_{+S}$ and average spreading velocity decrease with the increase in wedge angle at same time interval owing to the weaker capillary rising effect in FC (Figure 5b) [48]. An anisotropic liquid spreading factor is used to describe the unidirectional liquid spreading extent, which is defined as

(3) $$\eta =\frac{L_{+S}-L_{-S}}{\max\;\left(L_{+S},\;L_{-S}\right)}$$

As shown in the inset image in Figure 5b, the anisotropic factors of channels with different wedge angles are all close to 1, indicating the strong stability of the pinning effect. Furthermore, a PDMS block was placed in front of the liquid motion trajectory to observe the pinning failure process (Figure 5c). With the continuous injection of liquid, a single bulge is gradually formed in the middle of the channel until liquid effuses from the hydrophobic boundary, and the rear side of the liquid is pinned effectively by the pit’s edge in the whole process. These results indicated that the STC channels with biomimetic microcavities possess excellent unidirectional liquid transport capability.

#### 3.4.2. Influence of Microcavity Dimension

**Figure 6:**
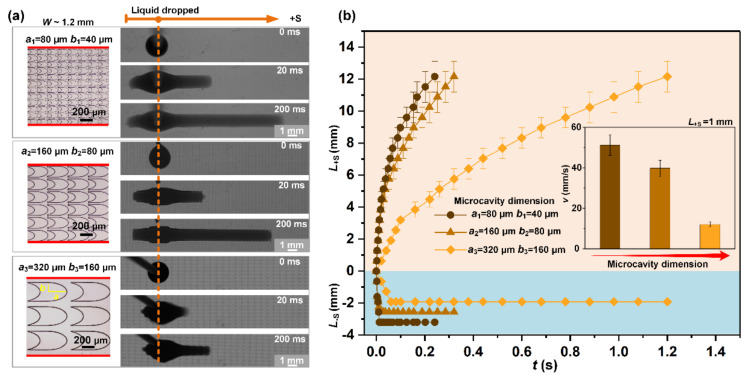
(**a**) Influence of the dimension of microcavity on unidirectional spreading. (**b**) Transport distance of liquid as a function of time. The inset image shows the average transport velocity of liquid on the channels with different microcavity dimensions.

Experiments were performed to further elucidate the influence of the pit’s dimensions. As optical images shown in the left side of Figure 6a, STC channels with the same hydrophilic stripe width (~1.2 mm) and proportional pit structures were constructed. The elliptical contour of the pit remains the same and the dimension ratio is 1:2:4. The semi-major axis length *a* of ellipse arc are 80, 160, and 320 μm, respectively; and semi-minor axis length *b* of ellipse arc are 40, 80, and 160 μm, respectively. Hence, the radii of curvature *R*_1_ in the proportional pits is twice and four times the value of that in pit structure with original size, then, a lower Laplace pressure is generated on the structure with larger pits. As the size of the pit increases, the spreading velocity of the liquid in the +S direction becomes significantly slower, decreasing from 51 ± 5 mm s^−1^ to 10 ± 2 mm s^−1^, on account of the speed of the liquid propagation process in the larger pits drops (Figure 6b). In the -S direction, although the wedge angle of pits is almost consistent, the channel with larger pits size shows stronger pinning ability due to the lower negative Laplace pressure, which corresponds to the above theoretical analysis. Therefore, in addition to the wedge angle and edge profile of microcavity, the dimension also plays an important role in the unidirectional liquid spreading control.

### 3.5. Application of STC Unidirectional Liquid Spreading Channel

#### 3.5.1. Spatial Unidirectional Liquid Spreading

**Figure 7:**
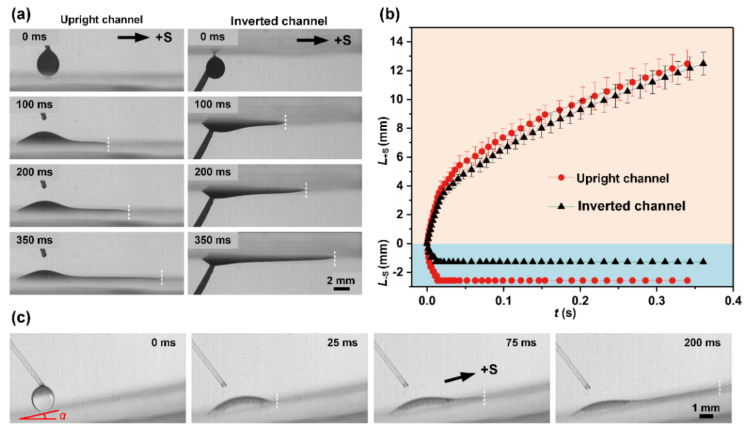
(**a**) Time-lapse images of liquid transport process on upward and downward channels. (**b**) Relative transport distance versus time for liquid spreading on the channels. (**c**) Spontaneous antigravity unidirectional liquid transport on the slope.

The STC unidirectional liquid spreading channel can not only induce liquid transport in horizontal paths, but also in spatial paths. As shown in Figure 7a, we fixed the channel surface on an inverted operation plate, and the droplet was dripped on the channel from bottom to top. It is noted that liquid could also transport spontaneously without external energy input, in which the spreading speed in the +S direction is almost the same as upright channel (Figure 7b). These results further illustrate that the gravitational potential energy could be negligible compared with the surface tension in the motion of liquid. Under the influence of droplet gravity, the liquid film thickens and the spreading distance in the −S direction is significantly shorter when the sample is upside down. Besides, spontaneous antigravity unidirectional transport is also reported here, which provides a new strategy for liquid control in 3D microfluidic systems. Liquid flows upward along the slope with ~10° inclination after dripping the droplet onto the bottom of the slope (Figure 7c). Within 200 ms, liquid can transport a distance of about 8 mm and elevates about 2.5 mm.

#### 3.5.2. Concise Applications in Microfluidic Devices and Microreactors

**Figure 8:**
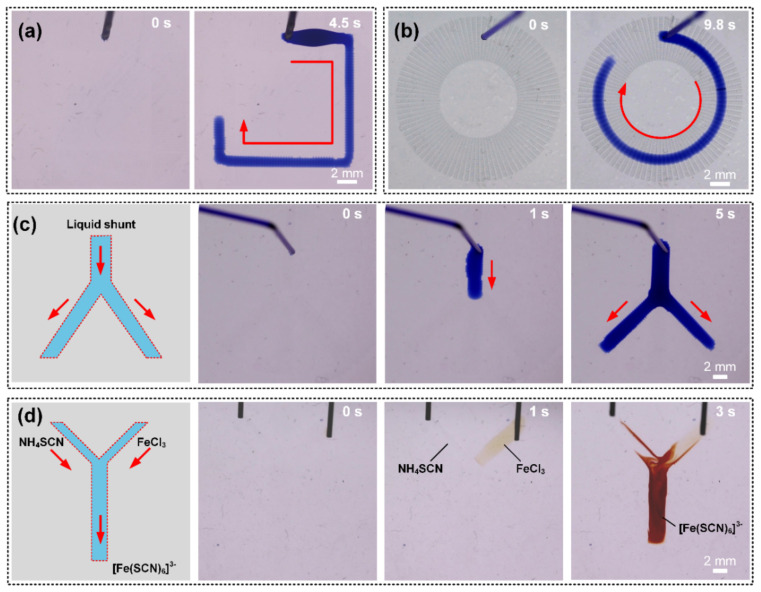
(**a**,**b**) Continuous unidirectional water transportation along predetermined rectangular and circular liquid motion trajectory. (**c**) Liquid shunt in Y-shaped branch channel. (**d**) Microreactor was performed between FeCl_3_ solution and NH_4_SCN solutions.

Microfluidics device has emerged as an efficient way for the development of biotechnology and point-of-care platforms [52,53,54]. For this purpose, a flexible, easy-fabricating, portable device with excellent controllability is strongly desired. Here, the STC unidirectional liquid spreading channel may offer new insight into the liquid manipulation. The time sequence images in Figure 8a,b demonstrate that the flexible liquid transport path can be realized easily by adjusting the arrangement of pits array and the hydrophilic pattern on hydrophobic substrate. With the continuous liquid injection, the liquid transports along predetermined motion path, which can provide a valuable solution to regulate liquid motion trail in open-microfluidic devices. In addition, a liquid shunt can also become reality on a Y-shaped channel containing one main channel and two arm channels. Liquid travels along the main channel firstly and then can be divided into two streams on the arm channels (Figure 8c). A proof-of-concept microreactor was constructed to further demonstrate the feasibility in lab-on-chips. The aqueous solutions of ammonium thiocyanate (NH_4_SCN, 0.5 M in H_2_O) and ferric chloride (FeCl_3_, 0.25 M in H_2_O) were deposited on the two arm channels by microsyringes, respectively. As shown in Figure 8d, a sharp color change from transparent to reddish brown occurs in main channel after mixing, due to the reaction products of ferric thiocyanate. A microreactor is therefore realized.

## 4. Conclusions

In summary, we proposed a strategy to realize excellent unidirectional liquid spreading on the basis of a chemical wettability pattern and physical biomimetic microstructures. The influence of design parameters on unidirectional liquid spreading was systematically explored. The channel width and microcavities dimension were proved to be essential for the liquid transport behavior, and a fast forward transport velocity can be achieved on the channel with wider hydrophilic stripe width and smaller textures. Moreover, the stability of the pinning effect was demonstrated and the reverse flow only happens in the initial stage under inertia action. Through analyzing the microscopic propagation process of liquid advancing edge and pinning effect, the underlying mechanism was revealed. Finally, arbitrary self-driven flow paths in 2D and 3D plane and chemical reactions were realized on the well-designed channels. This work could open an avenue to construct cost-effective unidirectional surfaces for microfluidics devices and microreactors.

## Supplementary Materials

The following are available online at https://www.mdpi.com/2072-666X/11/11/978/s1, Video S1: Unidirectional liquid transport behavior on STC channel (0.2× speed).
